# What, who and when? Incorporating a discrete choice experiment into an economic evaluation

**DOI:** 10.1186/s13561-016-0108-4

**Published:** 2016-07-29

**Authors:** Michela Tinelli, Mandy Ryan, Christine Bond

**Affiliations:** 1 LSE Health and Social Care, the London School of Economics and Political Science, Houghton Street, London, WC2A 2AE, UK; 2 Health Economics Research Unit, University of Aberdeen, Polwarth Building, Foresterhill campus, AB25 2ZD, Aberdeen, Scotland; 3 Centre of Academic Primary Care, University of Aberdeen, Polwarth Building, Foresterhill campus, AB25 2ZD, Aberdeen, Scotland

**Keywords:** Economic evaluation, Discrete choice experiment (DCE), Willingness to pay (WTP), Quality adjusted life year (QALY), Cost-benefit analysis (CBA), Randomised controlled trial (RCT)

## Abstract

**Background:**

Economic evaluation focuses on Quality-Adjusted-Life-Years (QALYs) as the main valuation method. However, it is well known that factors beyond health related quality of life are important to patients and the public. Whilst discrete-choice-experiments (DCE) have been extensively used to value such factors, their incorporation within an economic evaluation framework is limited. This study is the first to incorporate patient preferences for factors beyond QALYs into an economic evaluation and compare results with the standard cost-per-QALY approach, using randomised-controlled-trial (RCT) participants.

**Methods:**

Costings, clinical-effectiveness (appropriateness-of-treatment), QALYs and patient satisfaction data were collected at baseline and 12-month follow-up for a new pharmacy-service within a randomised-controlled-trial. Trial participants who replied to the follow-up survey and had not subsequently withdrawn from the study were mailed a DCE questionnaire at 24-months. WTP for the standard and new service was derived from the DCE. Results from QALYs and the DCE were compared.

**Results:**

At 12 months, costs, clinical-effectiveness and QALYs did not differ between the *intervention* and *control*; however there was a significant increase in satisfaction in the *intervention*. The DCE valued this increased satisfaction in the *intervention* (positive net-benefit). The longer the time patients experienced the new service the greater the reported net-benefit.

**Conclusion:**

When incorporating a DCE into an economic evaluation a number of questions are raised: *what* factors should be valued, *whose* values (trial-groups vs. all–trial-population) and *when* should they be elicited (still-receiving-the-intervention or afterwards). Consideration should also be given to status *quo* bias.

**Electronic supplementary material:**

The online version of this article (doi:10.1186/s13561-016-0108-4) contains supplementary material, which is available to authorized users.

## Background

Economic evaluation is an integral component of health care decision making. Whilst decision makers, such as the UK National Institute for Health and Care Excellence (NICE; http://www.nice.org.uk), the Scottish Medicine Consortium (SMC; http://www.scottishmedicines.org.uk), or the Canadian Agency for Drugs and Technologies in Health (CADTH; https://www.cadth.ca/), make policy recommendations based on ‘health outcomes’ (e.g. clinical-effectiveness; quality-adjusted-life-years, QALYs), the challenge remains to find a way of incorporating a broader measure of value, taking account of process utility and patient experiences into economic evaluations. Failure to do so may lead to incorrect policy conclusions.

Discrete Choice Experiments (DCEs) were introduced into health economics in the early 1990s to value aspects of health care beyond health outcomes [1, 2]. Since then, their use has increased to address a broad range of policy questions. Whilst many studies have generated monetary values from a DCE, the application of such values within an economic evaluation is limited, with only three studies identified. One study [3] compared DCE generated monetary values with QALYs, using different study samples. Two other studies ([4, 5]) included a DCE within a trial, but there was no comparable QALY data.

Despite their limited use in economic evaluations it is acknowledged that DCEs should be considered by policy makers and adopted as a useful tool when conducting evaluations of health care interventions [2, 6, 7]. The study reported here is the first, to our knowledge, to incorporate DCEs into an economic evaluation (using a cost benefit analysis, CBA) and compare results with the standard cost per QALY approach (using a cost-utility analysis, CUA), using the same trial participants. The application is a RCT looking at extending the role of the community pharmacist in the management of coronary heart disease. As well as comparing the results of a CBA and CUA analysis (*what* factors should be valued? Should we go beyond QALY?), consideration is given to: *whose* preferences should be elicited within a trial (patients with different experience of the service vs. overall sample of trial participants), and *when* (whilst still receiving the intervention or afterwards)?

## Methods

To address the question of *what* factors should be valued the QALY and DCE approach were conducted within the trial data using CUA and CBA respectively. To address the question of *who* and *when*, comparison of CUA and CBA was conducted at 3 levels:

- *All trial* patients (*who,* population with different levels of experience of the service being valued): in line with CUA analysis mean QALY in the population will be estimated and considered alongside costs, whereas for the CBA analysis, mean WTP in the population will be estimated and compared to costs;
- Across trial groups (who, patients with direct experience of the service being valued:– intervention - vs. patients with no experience of it - control): here it is assumed that the relevant QALY and WTP estimates come from the individuals that were part of the intervention group (because the control group may lack a firm understanding of the “new service”);
- Within intervention group (*when,* whilst still receiving the intervention or afterwards) - here the relevant QALY and WTP estimate are compared between intervention subgroups either still receiving or not receiving the *Medman service* at the time of the DCE survey (*intervention still receiving at 24 months* and *intervention not receiving at 24 months*). The former group refers to patients who continued to receive the *Medman service* after trial completion on a voluntary basis.

### The community pharmacy medicines management RCT (*Medman Trial)*

The *Medman Trial* was a multicentred RCT commissioned by the UK Department of Health to inform changes to the community pharmacist contractual framework. [8]. Subjects were male or female, aged 18 years and over, with recorded coronary heart disease (CHD), defined as previous myocardial infarction, angina, coronary artery by-pass graft, or angioplasty. Patients were identified from general practice computer systems, recruited and randomised (2:1) to *intervention* (receiving the *Medman service*) or *control* (receiving *usual care*). The *Medman service* was a collaborative medicine review service between the community pharmacist and the general practitioner (GP). It included an initial consultation with a community pharmacist to review: appropriateness of therapy (e.g. additional medicine required, medicine that should be discontinued, change of medicine, use of over-the-counter (OTC) medicines, formulation issues); compliance and concordance (e.g. daily consumption of medicine, any concerns/beliefs about medicines, information requirements); lifestyle (e.g. smoking cessation, increased exercise and dietary change); and social and support issues (either managing their medicines or their condition generally). The number of subsequent consultations was determined by the community pharmacist on the basis of each patient’s need, with a maximum of four anticipated during the 12-month follow-up. The community pharmacist communicated any suggested changes to prescription medicines to the GP using a standard referral form. The *control* patients received *usual care* from their community pharmacists (opportunistic advice on OTC medicines and lifestyle, and *ad hoc* communication with the GP) and GP (authorisation of repeat medicines, review of medicines).

The costing exercise collected information on NHS and patient costs, (2015 prices). More information on the costing is presented elsewhere [9]. A composite measure of ‘appropriate treatment’ was developed as a primary outcome [8]. The primary utility measures were EuroQol (EQ-5D; http://www.euroqol.org/) and SF-6D (http://www.shef.ac.uk/scharr/sections/heds/mvh/sf-6d) scores. Patient self-reported satisfaction was collected at 12 month follow-up. A satisfaction score was developed to include experience of and satisfaction with their most recent community pharmacy visit and was assessed by measuring response to 15 positive and negative statements.. [10]. All data were collected by patient completed postal surveys at baseline and 12-month follow-up; differences between baseline and 12-month follow-up were compared using a paired *t* test.

### Discrete choice experiment

#### Developing and distributing the DCE

Attributes and levels for the DCE were informed by the aims of the *Medman service*, that is - to increase the chance of receiving the most appropriate treatment; previous studies conducted in the pharmacy setting [11–13]; and analysis of the Medman patient survey data, using Exploratory Factor Analysis (EFA) [14]. Specifically, questions describing respondent attitudes and expectations were grouped into summary factors: type of service provided by the pharmacist; the pharmacy premises; patient-pharmacist relationship; a wider advisory health-related role for the pharmacist; a traditional dispensing role for the pharmacist [15]. A ‘cost’ (price proxy) attribute was included so that WTP, a monetary measure of outcome, could be estimated. The final set of attributes and levels are shown in Tables 1 and 2, more detail is provided elsewhere [15].

**Table 1:** Medman trial data for respondents to the DCE survey

		All Medman trial participants (Intervention and control combined; *N* = 554)		*P* value^a^
		Baseline	12-month follow-up	
Costs				
Costs	mean	1410.9	1422.895	0.85
	SD	1252.87	1199.32	
Difference in costs			11.995	
			448.22	
Outcomes				
Appropriate treatment score	mean	4.25	4.33	0.11
	SD	0.905	1.02	
SF-6D Score	mean	0.72	0.72	0.99
	SD	0.14	0.15	
EQ-5D Score	mean	0.73	0.74	0.45
	SD	0.24	0.265	
Satisfaction score	mean	42.63	46.52	<0.01
	SD	9.67	16.55	

^a^Differences between baseline and 12-month follow-up were tested using paired *t* test statistics

**Table 2:** Medman trial data for respondents to the DCE survey (continued)

		Trial groups						Intervention subgroups					
		Intervention *N* = 364		P val^a^	Control *N* = 190		P val^a^	Still receiving at 24 months *N* = 188		P val^a^	Not receiving at 24 months *N* = 176		*P* val^a^
		Baseline	12-month follow-up		Baseline	12-month follow-up		Baseline	12-month follow-up		Baseline	12-month follow-up	
Costs													
			559.12			336.23			327.00			303.40	
Costs	mean	1579.40	1568.20	0.90	1242.40	1277.59	0.68	1653.94	1617.20		1499.77	1515.85	
	SD	1323.11	1472.54		1182.63	926.10		1299.74	1510.42		1346.76	1433.45	
Difference in costs			−11.20			35.20			−36.73	0.77		16.08	0.89
			559.65			336.79			325.25			303.32	
Outcomes													
Appropriate treatment score	mean	4.20	4.26	0.55	4.30	4.40	0.51	4.15	4.27	0.44	4.25	4.25	1.00
	SD	0.94	1.03		0.87	1.01		0.97	0.94		0.92	1.12	
SF-6D Score	mean	0.71	0.71	0.55	0.73	0.73	0.66	0.70	0.69	0.14	0.72	0.73	0.28
	SD	0.14	0.15		0.14	0.15		0.14	0.14		0.14	0.15	
EQ-5D Score	mean	0.73	0.74	0.11	0.73	0.74	0.24	0.73	0.71	0.59	0.73	0.74	0.28
	SD	0.24	0.26		0.24	0.27		0.23	0.27		0.26	0.25	
Satisfaction score	mean	42.36	48.30	<0.01	42.90	44.74	0.86	45.74	52.38	0.02	39.26	43.67	<0.01
	SD	9.89	15.75		9.45	17.34		9.68	15.78		9.07	14.49	

^a^Differences between baseline and 24-month follow-up were tested using paired *t* test statistics

**Fig. 1:**
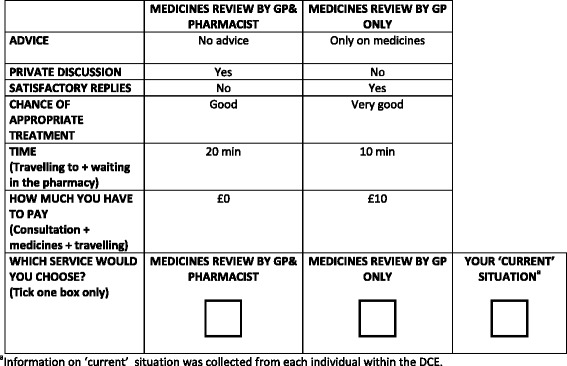
Example of DCE question

A labelled design was employed for the DCE using ‘*Medicines review by GP & pharmacist*’ (GP and PH: the *Medman service* received by the *intervention*) and ‘*Medicines review by GP only*’ (GP: the *usual care* received by the *control*) labels. An orthogonal labelled 32-choice set design was created from design catalogues using foldover methods [16], and subsequently allocated into four blocks of eight choices using appropriate software [17]. A ‘current’ situation option was added to each choice set with levels for the attributes collected in the questionnaire. Two contraction tests were included to test the validity of responses [18]^1^. Responses were excluded when individuals failed both tests; failing one test only was considered as random error. An example of a choice question is presented in Fig. 1.

The DCE questionnaire also collected respondent’s socioeconomic and demographic information. Ethics approval for the study was granted from the MREC for Scotland as an amendment to previous approval for conducting the *Medman Trial* [8].

Pilot work with 312 patients attending two general practice clinics confirmed validity of the questionnaire (about 90 % of respondents passed both contraction tests) and its ability to be completed (respondents reported a median of 3 on a 5-point Likert scale from 1 (not difficult) to 5 (very difficult)). The final DCE survey was mailed 24 months after the baseline survey to all trial participants who had replied to the 12-month follow-up survey. *Intervention* patients were asked if they had continued to receive the *Medman service* from their pharmacist since the formal end of the *Medman Trial* 12 months previously.

#### DCE analysis

The Hausman test showed that the IIA assumption held across groups and the conditional logit (CL) model was subsequently used to analyse response data, with the following function estimated:

1 $$ {V}_{ji} = AS{C}_j + {\beta}_1 ADVMED + {\beta}_2 ADVHL + {\beta}_3 ADVMH + {\beta}_4 TIME + {\beta}_5 PRIVD + {\beta}_6 REPLD + {\beta}_7CH0 + {\beta}_8CH1 + {\beta}_9CH2 + {\beta}_{10} COST $$

V_ij_ is the systematic part of the utility function observable by the researcher of the jth choice to the ith individual, and ε_ji_ is the error term. Dummy variables were used to analyse categorical attributes, with reference levels identified, and listed in Table 3, together with definitions of all labels. ASC_j-1_ is the Alternative Specific Constant term, describing the general preference for GP&PH (ASC_GP_&_PH_) or GP (ASC_GP_), over the ‘current’ situation (with reference level dummy variables captured in these constants). β_1_-β_10_ are the coefficients to be estimated.

**Table 3:** Alternatives, attributes and levels for the DCE

Defining the alternatives	Alternative Names	
The Community Pharmacy Medicines Management Project Evaluation Team (8); Tinelli et al. (13) Medman patient survey data, analysed using Exploratory Factor Analysis (15)	- Medicines review by GP & pharmacist (GP&PH) - Medicines review by GP only (GP) - ‘Current’ situation	
Defining the attributes/levels	Attribute name	Levels and regression coding
Ryan et al. (11); Payne et al. (12) Medman patient survey data, analysed using Exploratory Factor Analysis (15)	‘Advice’ [advice on medicines & health/ lifestyle]	- No advice ^a^ - Only on medicines (ADVMED) - Only on health/lifestyle (ADVHL) - On medicines & health/lifestyle (ADVMH)
Payne et al. (12) Medman patient survey data, analysed using Exploratory Factor Analysis (15)	‘Privacy’ in the pharmacy [consultation with your pharmacist is in a private area]	- No ^a^ - Yes (PRIVD)
Tinelli et al. (13); Payne et al. (12) Medman patient survey data, analysed using Exploratory Factor Analysis (15)	‘Time’ [time spent travelling to, waiting in the pharmacy]	10 min; 20 min; 30 min; 40 min (TIME)
Medman patient survey data, analysed using Exploratory Factor Analysis (15)	‘Satisfactory replies’ to your questions	- No ^a^ - Yes (REPLD)
The Community Pharmacy Medicines Management Project Evaluation Team (8); Tinelli et al. (13)	‘Chance’ of receiving appropriate treatment	- Very poor (CH0) - Poor (CH1) - Good (CH2) - Very good ^a^
Ryan et al. (11); Payne et al. (12); Tinelli et al. (13)	‘Cost’; How much you have to pay (as an indicator of value) [cost of the medicine + the cost of the medicines review and advice received + the cost of any travel]	£0; £10; £20; £30 (COST)

^a^ Reference level

The welfare change in moving from the control situation (‘*Medicines Review by GP only)*’ to the intervention situation (‘*Medicines review by GP & pharmacist)*’ was estimated^2^ [19] and incorporated into a CBA framework [20, 21]. Trial costs derived at baseline and 12-month follow-up (see Tables 1 and 2) were used. It was assumed that there was no change in patient preferences between 12 and 24-month follow-up. Net-benefit (NB, WTP monetary benefits – monetary costs) estimates across groups were calculated. Differences in WTP and NB estimates were tested across groups using appropriate tests (chi squared test for categorical data; independent *t* test statistics for continuous normally distributed data and Mann–Whitney test statistics for continuous not normally distributed data). The DCE data were modelled using NLOGIT 4.0, whereas other analyses where undertaken using IBM SPSS statistics 21.0.A series of mixed logit (MXL) models were included in the analysis to allow for unobserved heterogeneity across individuals.  Different model specifications were compared using the log likelihood ratio index (LLRI) and the preferred MXL (with highest LLRI) was employed to inform the WTP estimates (see Additional file 1); its attached WTP estimates did not change the direction of the CBA findings based on the CL model (see Additional file 2).

## Results

### The community pharmacy medicines management RCT (*Medman Trial) – a cost utility analysis*

Tables 1 and 2 shows results for the subset of respondents who provided 12-month follow up data and completed the DCE (see below). No statistically significant difference was observed across groups in costs, appropriate treatment score or QALYs. However, satisfaction increased for *all Medman Trial* patients and the *intervention* patients, but not in c*ontrol* patients. The DCE aimed to value this increased satisfaction.

### Discrete choice experiment – a cost-benefit analysis

Patients’ responses, their characteristics and their ‘current’ situation at 24-month follow-up are shown in Table 4. 94 % (441/469) of respondents from the *intervention* and 96 % (235/245) of respondents from *control* passed the two contraction tests. Data eligible for analysis, passing the two contraction tests and including ‘current’ situation data, came from 78 % of the respondents (*All Medman Trial* 78, *intervention* 78, *control* 78; *intervention still receiving at 24 months* 76 and *intervention not receiving at 24 months* 79 %). The *intervention* group had similar characteristics and ‘current’ experience compared with the *control* group. The *Intervention still receiving at 24 months* sub-group patients were significantly older and had lower income compared with *intervention not receiving at 24 months* (*p* < 0.01). Overall the former reported more frequently that their ‘current’ situation was characterized by receiving ‘advice’ (*p* < 0.01), ‘privacy’ of the premises (*p* < 0.01), receiving ‘satisfactory replies’ (*p* < 0.01), shorter waiting ‘time’ (*p* < 0.03) or decreased ‘costs’ (*p* < 0.03), and less favorable ‘chance’ of receiving appropriate treatment (*p* < 0.01).

**Table 4:** DCE patient survey respondents: DCE responses, individual characteristics and ‘current’ situation at 24-month follow-up

	All Medman trial participants	Trial groups			Intervention subgroups		
	Intervention and control together	Intervention	Control		Intervention still receiving at 24 months	Intervention not receiving at 24 months	
	N (%)^a^	N (%)^a^	N (%)^a^	P value^b^	N (%)^a^	N (%)^a^	P value^b^
DCE completed	714 (100)	469 (100)	245 (100)	-	247 (100)	222 (100)	-
DCE passing rationality tests	676 (95)	441 (94)	235 (96)	-	232 (94)	208 (94)	-
DCE eligible for analysis^c^	554 (78)	364 (77.8)	190 (77.5)	-	188 (76.1)	176 (79.3)	-
Gender (male)	387 (54)	250 (69)	137 (72)	0.40	125 (66)	125 (71)	0.35
Age (mean (SD))	70.595 (8.31)	70.44 (8.53)	70.75 (8.09)	0.67	72.00 (8.23)	68.77 (8.55)	<0.01
*Income*				0.08			<0.01
-Up to £20,000	366 (51)	249 (79)	117 (71)		152 (87)	97 (69)	
-£20,000-£40,000	100 (14)	57 (18)	43 (26)		20 (11)	37 (26)	
-Over £40,000	14 (2)	9 (3)	5 (3)		2 (1)	7 (5)	
‘Current’ situation							
*Advice*				0.98			<0.01
- No advice	230 (32)	150 (41)	80 (42)		36 (19)	114 (65)	
-On medicines only	175 (25)	114 (31)	61 (32)		79 (42)	35 (20)	
- On health only	9 (1)	6 (2)	3 (2)		1 (1)	5 (3)	
-On Medicines/Health/life	140 (20)	94 (26)	46 (24)		72 (38)	22 (13)	
Privacy (yes)	308 (43)	215 (59)	93 (49)	0.23	146 (78)	69 (39)	<0.01
Replies (yes)	267 (37)	186 (51)	81 (43)	0.06	130 (69)	56 (32)	<0.01
*Chance*				0.34			<0.01
-Very poor	14 (2)	9 (2)	5 (3)		3 (2)	6 (3)	
-Poor	204 (29)	140 (39)	64 (34)		93 (50)	47 (27)	
-Good	297 (42)	190 (52)	107 (56)		88 (47)	102 (58)	
-Very Good	36 (5)	23 (6)	13 (7)		3 (2)	20 (11)	
Time (median [IQR])	15 [10–30]	15 [10–30]	15 [10–30]	1	15 [10–25]	17.5 [10–30]	0.03
Cost (median [IQR])	0 [0–0.58]	0 [0–0.58]	0 [0–0.8]	0.53	0 [0–0.4]	0 [0–0.8]	0.03

^a^ Percentages refer to the respondents completing each question; ^b^ Differences between groups at 24-month follow-up were tested using: independent *t* test statistics (continuous normally distributes data); Mann–Whitney test (continuous not normally distributed data); chi-squared test (categorical data); ^c^ Valid responses passing rationality testing and completing answers on their ‘current’

Comparison of models between groups (Table 5) showed that at an aggregate level the ‘current’ situation was always preferred to the alternatives of ‘*Medicines review by GP & pharmacist*’ or ‘*Medicines review by GP only*’ (*p* < 0.01; all groups). Respondents also preferred: ‘satisfactory replies’ (Compared to no reply; *p* < 0.01 *all Medman trial, intervention* and its subgroups); very good ‘chance’ of receiving appropriate treatment (compared with very poor/poor; *p* < 0.10 all groups apart from good ‘chance’ of receiving appropriate treatment in the i*ntervention*, and poor ‘chance’ of receiving appropriate treatment in the *control*)); and lower costs (*p* < 0.05; all groups). *All Medman trial, intervention* and *intervention still receiving* groups valued receiving ‘advice’ on medicines only (compared with no advice; *p* < 0.01).

WTP values are shown in Table 6 when moving from ‘*Medicines review by GP only*’ (*usual care*) to ‘*GP and Pharmacist collaboration on in Medicines review’* (*Medman service*) as follow:

**Table 5:** DCE regression results

	All Medman trial participants			Trial groups						Intervention subgroups					
	Intervention and control together			Intervention			Control			Intervention still receiving at 24 months			Intervention not receiving at 24 months		
	Coeff.	SE	P-val	Coeff.	SE	P-val	Coeff.	SE	P-val	Coeff.	SE	P-val	Coeff.	SE	P-val
(Compared to no advice)															
‘Advice’ on medicines only	0.154	.052	<0.01	0.169	0.065	<0.01	0.138	0.090	0.125	0.389	0.095	<0.01	0.126	0.100	0.204
‘Advice’ on health/lifestyle only	−0.009	0.069	0.89	0.029	0.086	0.734	−0.069	0.121	0.568	0.197	0.123	0.109	−0.009	0.126	0.945
‘Advice’ on medicines & health/lifestyle	0.019	0.055	0.721	0.074	0.068	0.276	−0.088	0.095	0.358	0.214	0.096	<0.05	0.184	0.107	0.087
(Compared to no privacy)															
‘Privacy’ in the pharmacy	-.016	.040	.679	0.007	0.050	0.891	−0.053	0.071	0.453	−0.010	0.071	0.893	0.126	0.074	0.090
(Compared to no reply)															
‘Satisfactory replies’ to your questions	.142	.040	<0.01	0.151	0.050	<0.01	0.134	0.071	0.058	0.197	0.068	<0.01	0.193	0.075	<0.01
(Compared to very good)															
Very poor ‘chance’ of receiving appropriate treatment	-.365	.069	<0.01	−0.371	0.086	<0.01	−0.360	0.121	<0.01	−0.466	0.121	<0.01	−0.262	0.125	<0.05
Poor ‘chance’ of receiving appropriate treatment	-.222	.063	<0.01	−0.255	0.078	<0.01	−0.163	0.109	0.134	−0.244	0.111	<0.05	−0.209	0.114	0.066
Good ‘chance’ of receiving appropriate treatment	-.155	.059	<0.01	−0.083	0.073	0.255	−0.312	0.106	<0.01	−0.083	0.106	0.433	−0.055	0.104	0.595
TIME	-.003	.002	.116	−0.003	0.002	0.101	−0.002	0.003	0.600	−0.001	0.003	0.646	−0.007	0.003	<0.05
COST	-.008	.002	<0.01	−0.009	0.003	<0.01	−0.007	0.004	0.080	−0.010	0.004	0.013	−0.009	0.004	<0.05
(compared to current)															
Medicines review by GP & Pharmacist^a^	−1.128	.059	<0.01	−0.990	0.071	<0.01	−1.420	0.108	<0.01	−0.640	0.101	<0.01	−1.443	0.110	<0.01
Medicines review by GP only^a^	-.994	.057	<0.01	−0.997	0.070	<0.01	−1.013	0.101	<0.01	−0.943	0.105	<0.01	−1.057	0.103	<0.01
No of observations	4445			2916			1529			1501			1415		
No of individuals	554			364			190			188			176		

^a^Everything else constant

**Table 6:** WTP estimates (£) at 24-month follow-up and Net Benefit analysis

		All Medman trial participants			Trial groups			Intervention subgroups		
		Intervention and control together	P-val^a^ Vs. Intervention	P-val^a^ Vs. Control	Intervention	Control	P-val^a^	Intervention still receiving at 24 months	Intervention not receiving at 24 months	P-val^a^
Willingness to pay (WTP) when moving from ‘Medicines review by GP only’ to ‘Medicines review by GP & Pharmacist’	mean	−26.475	<0.01	<0.01	3.52	−56.47	<0.01	41.55	−34.25	<0.01
	SD	10			1.17	18.83		13.85	11.42	
Difference in societal costs from Tables 1 and 2		11.99			−11.20	+35.20		−36.73	+16.08	
Net Benefit (NB)^b^	mean	−38.47	0.44	0.30	14.72	−91.67	<0.01	78.29	−50.33	<0.01
	SD	229.11			1620.60	1167.68		1706.09	1528.41	

^a^ Differences between groups at 24-month follow-up were tested using Mann–Whitney test statistics. ^**b**^ NB was calculated as: (WTP at 24-month follow-up, from DCE patient survey data; see Table 5) – (difference in society costs, from Medman Trial data see Tables 1 and 2)

- *All trial* patients (*who,* population with different levels of experience of the state being valued): *All Medman Trial* participants reported a negative WTP value of -£26.48; this was significantly different from both *intervention* and *control* group.
- *Across trial* groups (*who,* patients with direct experience of the state being valued – *intervention* – vs. patients with no experience of it - *control*): The *intervention* group preferred the *Medman service* to *usual care (£3.52) whereas the control* group did not value the change, with a negative value (−£-56.47). This difference was statistically significant (*p* < 0.01).
- Within the *intervention* subgroups (*when,* whilst still receiving the intervention or afterwards): Those experiencing the *Medman service* for 24 months valued the service at £41.55; in comparison those who experienced the *Medman service* for a shorter period (*intervention not receiving at 24 months*) preferred *usual care* (−£34.25). This difference was statistically significant (*p* < 0.01). Prolonged experience of the *Medman service,* beyond the *Medman Trial,* had a positive impact on value.

Combining WTP values with costs, NB estimates show:

- *All trial* patients (*who,* population): Negative values for the *All Medman Trial* (comprising extra cost and negative benefits). The NB for the *all Medman trial* participants was negative (−£14.48) and it was statistically significant different from the *control* (−£91.67; *p* < 0.01) and the intervention groups (£14.72; *p* < 0.01).
- *Across trial* groups (*who,* patients): The *intervention* reported a reduction in costs at follow-up as well as positive mean WTP values. It followed that a positive NB for the *Medman service* was reported (£14.72 *intervention* compared to -£91.67 *control*, *p* < 0.01).
- Within the *intervention* subgroups (*when*): Prolonged experience of the *Medman service,* beyond the *Medman Trial,* had a positive impact on NB estimates (£78.29 *intervention still receiving at 24 months* compared to -£50.33 *intervention not receiving at 24 months, p* < 0.01).

## Discussion

The results provide evidence that the DCE approach is able to value increased satisfaction. This is consistent with the extensive health economics literature suggesting values from the process utility benefits, using discrete choice experiments and contingent valuation [1, 2, 22, 23].

This findings also raises the question of *whose* values should be elicited in an economic evaluation, and *when*.

*Whose* values should be elicited in an economic evaluation? Our study elicited *patient values*, assuming patients themselves should be asked to value their own health state given that they have direct experience of the state being valued, and compared results from all trial population and trial groups with different experience of the service. It could be argued that the relevant QALY and WTP estimates should come from the individuals that were part of the intervention group because the control group may lack a firm understanding of the new Medman service.

This paper goes further, arguing that patient values beyond health outcomes should be incorporated into an economic evaluation (*what* factors should be valued? Should we go beyond QALY?). Internationally, policy makers have recognised the importance of considering patients’ experiences. For example, the World-Health-Organization (WHO; http://www.who.int) considers that service ‘responsiveness’ describes how a healthcare system meets people’s needs, the Institute-of-Medicine (IOM; http://www.nationalacademies.org/) identifies ‘patient centered care’ as a key dimension of healthcare quality, and many national governments and healthcare organisations include these or similar concepts in their decision making (e.g. see NHS confederation covering NHS policy developments in UK and Europe, http://www.nhsconfed.org).

*When* should values be elicited? When considering the standard CBA approach comparing *intervention* and *control* groups the negative net benefit does not support the introduction of the intervention (extending the role of the pharmacist); however if we focus on the *intervention* group, results are supportive, but only for those *still receiving the intervention at 24 months*. This is not surprising as these individuals preferred the Medman service and decided to continue beyond 12-month trial follow-up (pending on the availability of the community pharmacist and the GP joint agreement to continue its delivery). It may be argued that the analysis within the intervention group may not be informative for the purpose of the economic evaluation and findings from the standard CBA approach comparing trial *intervention* and *control* should be preferred to inform policy.

The WTP suggested *status quo* bias [1], with those *still receiving the intervention at 24 months* preferring it compared to those who had not experienced it. Further, length of time experiencing the *intervention* had a significant positive effect on WTP, perhaps providing more evidence of *status quo* bias. People tend to follow the status quo when making health-related decisions and they most value the service they experience.

To our knowledge this is the first study to incorporate DCE WTP estimates into an economic evaluation and compare results with a CUA (clinical-effectiveness and QALYs). A limitation is that the comparison was not contemporaneous, and there is no data on the clinical and QALY measures at the 24 month point. Nonetheless the study adds to the limited CBA applications available within the health care literature, and informs current discussion on whether the choice of evaluation approach can impact on adopting decisions. It also raises issues that are important in this future and important research agenda.

## Conclusion

Policy decision making requires consideration of patient preferences and, moreover, the information on preferences can be used to develop effective delivery of community pharmacy service. However, findings from this study suggest when incorporating a discrete choice experiment into a trial economic evaluation, important issues that need to be addressed include: *what* factors should be valued, *whose* values (and *when*) should be elicited, and *status quo* bias.

## Additional files

Additional file 1: Table S1. Mixed logit model. (DOC 67 kb) Additional file 2: Table S2. Respondents participating in the DCE: WTP estimates (£) at 24-month follow-up and NB analysis (using results from mixed logit model). (DOC 34 kb)

## Footnotes

1 The rationality tests applied Sen’s contraction test. Here, individuals were initially presented with a choice set involving three choices (“Medicines review by GP& pharmacist”, “Medicines review by GP only” and “Your ‘current’ situation”). This choice was then repeated later in the questionnaire, with the choice set contracted to two options (“Medicines review by GP only”, and “Your ‘current’ situation”). These two options depended on what they chose initially, with the option they chose and one of the other options from the original three choices being presented. The individual should choose the same option when the choice set is contracted.

2 A state of the world model was assumed.
