# Supplementary material for: What, who and when? Incorporating a discrete choice experiment into an economic evaluation
Source: Health Econ Rev. 2016 Jul 29;6:31. doi: 10.1186/s13561-016-0108-4 (PMC4967060; doi:10.1186/s13561-016-0108-4)
Supplement: Additional file 1: Table S1. — Mixed logit model. (DOC 67 kb) [file 13561_2016_108_MOESM1_ESM.doc]

**Additional file 1: Table S**1. Mixed logit model

|  | **Trial groups** | | | | | | **Intervention subgroups** | | | | | | | **All Medman trial participants** | | | | |
| --- | --- | --- | --- | --- | --- | --- | --- | --- | --- | --- | --- | --- | --- | --- | --- | --- | --- | --- |
|  | **Intervention** | | | **Control** | | | **Intervention still receiving at 24months** | | | **Intervention not receiving at 24months** | | | | **Intervention and control together** | | | | |
| **Random parameters in utility functions and their standard deviations of normal distribution** | | | | | | | | | | | | | | | | | | |
|  | **Coeff.** | **SE** | **P-val** | **Coeff.** | **SE** | **P-val** | **Coeff.** | **SE** | **P-val** | **Coeff.** | **SE** | | **P-val** | **Coeff.** | **SE** | | | **P-val** |
| (Compared to Very good) |  |  |  |  |  |  |  |  |  |  |  | |  |  |  | | |  |
| **CH0** | -.765 | .269 | **<0.01** | -.406 | .180 | **.024** | -1.376 | .642 | **.032** | -.391 | .239 | | .102 | -.551 | .173 | | | **<0.01** |
| **SD** | 1.250 | .462 | **<0.01** | .339 | .685 | .620 | 2.033 | .883 | **.021** | .702 | .575 | | .221 | .812 | .386 | | | **.035** |
| (Compared to no reply) |  |  |  |  |  |  |  |  |  |  |  | |  |  |  | | |  |
| **ADVMH** | .044 | .076 | .561 | -.193 | .118 | .102 | .221 | .110 | **.046** | .183 | .109 | | .093 | -.027 | .063 | | | .659 |
| **SD** | .659 | .266 | **<0.01** | 1.148 | .276 | **<0.01** | .809 | .358 | .023 | .051 | .533 | | .922 | .801 | .188 | | | **<0.01** |
| **Non-random parameters in utility functions** | | | | | | | | | | | | | | | | | | |
|  | **Coeff.** | **SE** | **P-val** | **Coeff.** | **SE** | **P-val** | **Coeff.** | **SE** | **P-val** | **Coeff.** | **SE** | | **P-val** | **Coeff.** | **SE** | | | **P-val** |
| (Compared to no advice) |  |  |  |  |  |  |  |  |  |  |  | |  |  |  | | |  |
| **ADVMED** | .178 | .069 | **<0.01** | .139 | .093 | .135 | .439 | .106 | **<0.01** | .126 | .101 | | .211 | .157 | .054 | | | **<0.01** |
| **ADVHL** | .028 | .090 | .755 | -.074 | .127 | .561 | .211 | .136 | .122 | -.011 | .127 | | .929 | -.008 | .072 | | | .905 |
| (Compared to no privacy) |  |  |  |  |  |  |  |  |  |  |  | |  |  |  | | |  |
| **PRIVD** | .00003 | .054 | .994 | -.041 | .075 | .581 | -.010 | .080 | .900 | .120 | .076 | | .115 | -.013 | .043 | | | .756 |
| (Compared to no reply) |  |  |  |  |  |  |  |  |  |  |  | |  |  |  | | |  |
| **REPLD** | .1639 | .053 | **<0.01** | .148 | .075 | **.049** | .223 | .076 | **<0.01** | .196 | .076 | | **<0.01** | .151 | .042 | | | **<0.01** |
| (Compared to Very good) |  |  |  |  |  |  |  |  |  |  |  | |  |  |  | | |  |
| **CH1** | -.272 | .081 | **<0.01** | -.150 | .115 | .195 | -.305 | .120 | **<0.01** | -.208 | .114 | | .068 | -.229 | .065 | | | **<0.01** |
| **CH2** | -.093 | .075 | .218 | -.329 | .112 | **<0.01** | -.115 | .112 | .302 | -.058 | .104 | | .578 | -.161 | .062 | | | **<0.01** |
| **TIME** | -.003 | .002 | .087 | -.002 | .003 | .524 | -.001 | .003 | .713 | -.006 | .003 | | **.030** | -.003 | .001 | | | .098 |
| **COST** | -.009 | .003 | **<0.01** | -.007 | .004 | **.091** | -.010 | .004 | **<0.01** | -.009 | .004 | | **.027** | -.008 | .002 | | | **<0.01** |
| (compared to current) |  |  |  |  |  |  |  |  |  |  |  | |  |  |  | | |  |
| **ASC_GPPH** | -1.05 | .079 | **<0.01** | -1.540 | .122 | **<0.01** | -.689 | .116 | **<0.01** | -1.457 | .113 | | **<0.01** | -1.190 | .066 | | | **<0.01** |
| **ASC_GP** | -1.04 | .079 | **<0.01** | -1.097 | .112 | **<0.01** | -1.020 | .124 | **<0.01** | -1.064 | .104 | | **<0.01** | -1.045 | .063 | | | **<0.01** |
| **No of observations and goodness of fit** | | | | | | | | | | | | | | | | | | |
| **No of observations** | 2916 |  |  | 1529 |  |  | 1982 |  |  | 1415 | |  |  | 4445 | |  |  | |
| **No of individuals** | 364 |  |  | 190 |  |  | 188 |  |  | 176 | |  |  | 554 | |  |  | |
| **Log likelihood** | -2690.298 |  |  | -1349.499 |  |  | -1973.90 |  |  | -1229.025 | |  |  | -4057.201 | |  |  | |
| **LRRI** | 0.15 |  |  | 0.19 |  |  | 0.09 |  |  | 0.205 | |  |  | 0.167 | |  |  | |
