# Supplementary material for: What, who and when? Incorporating a discrete choice experiment into an economic evaluation
Source: Health Econ Rev. 2016 Jul 29;6:31. doi: 10.1186/s13561-016-0108-4 (PMC4967060; doi:10.1186/s13561-016-0108-4)
Supplement: Additional file 2: Table S2. — Respondents participating in the DCE: WTP estimates (£) at 24-month follow-up and NB analysis (using results from mixed logit model). (DOC 34 kb) [file 13561_2016_108_MOESM2_ESM.doc]

**Additional file 2: Table S2. Respondents participating in the DCE: WTP estimates (£) at 24-month follow-up and NB analysis (using** **results from mixed logit model)**

|  |  | **Trial groups** | | | **Intervention subgroups** | | | **All Medman trial participants** | | |
| --- | --- | --- | --- | --- | --- | --- | --- | --- | --- | --- |
|  |  | **Intervention** | **Control** | **P-val**^a^ | **Intervention still receiving at 24months** | **Intervention not receiving at 24months** | **P-val**^a^ | **Intervention and control together** | **P-val^a^ Vs. Intervention** | **P-val^a^ Vs. Control** |
| **Willingness to pay (WTP)**  **when moving from ‘Medicines review by GP only’ to ‘Medicines review by GP & Pharmacist ’** | **mean** | 1.01 | -2.48 | <0.01 | 11.06 | -2.05 | <0.01 | -1.62 | <0.01 | <0.01 |
|  | **SD** | 0.01 | 0.02 |  | 0.1 | 0.02 |  | 0.76 |  |  |
| **Difference in societal costs from Table 1** |  | -11.20 | +35.20 |  | -36.73 | +16.08 |  | 11.99 |  |  |
| **Net Benefit (NB)^b^** | **mean** | 12.21 | -37.68 | 0.02 | 47.79 | -18.13 | 0.18 | -13.615 |  | 0.27 |
|  | **SD** | 146.52 | 444 |  | 624 | 228 |  | 204 |  | <0.01 |

^a^ Differences between groups at 24-month follow-up were tested using Mann-Whitney test statistics. **^b^** NB was calculated as: (WTP at 24-month follow-up, from DCE patient survey data; see table 4) – (difference in society costs, from Medman Trial data see Table 1).
